# Metabolic engineering of *Priestia megaterium* for 2’-fucosyllactose production

**DOI:** 10.1186/s12934-024-02620-w

**Published:** 2025-01-04

**Authors:** Bu-Soo Park, Jihee Yoon, Jun-Min Lee, Sang-Hyeok Cho, Yoojeong Choi, Byung-Kwan Cho, Min-Kyu Oh

**Affiliations:** 1 Department of Chemical & Biological Engineering, Korea University, Seoul, 136-763 Korea; 2 Samyang Corp., 295 Pangyo-ro, Bundang-gu, Seongnam-si, Gyeonggi-do 13488 Republic of Korea; 3 Department of Biological Sciences, Korea Advanced Institute of Science and Technology, Daejeon, 34141 Republic of Korea; 4 KAIST Institute for the BioCentury, Korea Advanced Institute of Science and Technology, Daejeon, 34141 Republic of Korea; 5 Graduate School of Engineering Biology, Korea Advanced Institute of Science and Technology, Daejeon, 34141 Republic of Korea

**Keywords:** Metabolic engineering, 2’-fucosyllactose, α-1,2-fucosyltransferase, HMO, GRAS, *Priestia megaterium* ATCC 14581

## Abstract

**Background:**

2′-Fucosyllactose (2′-FL) is a predominant human milk oligosaccharide that significantly enhances infant nutrition and immune health. This study addresses the need for a safe and economical production of 2’-FL by employing Generally Recognized As Safe (GRAS) microbial strain, *Priestia megaterium* ATCC 14581. This strain was chosen for its robust growth and established safety profile and attributing suitable for industrial-scale production.

**Results:**

The engineering targets included the deletion of the *lacZ* gene to prevent lactose metabolism interference, introduction of α-1,2-fucosyltransferase derived from the non-pathogenic strain, and optimization of the GDP-L-fucose biosynthesis pathway through the overexpression of *manA* and *manC*. These changes, coupled with improvements in lactose uptake and utilization through random mutagenesis, led to a high 2’-FL yield of 28.6 g/L in fed-batch fermentation, highlighting the potential of our metabolic engineering strategies on *P. megaterium*.

**Conclusions:**

The GRAS strain *P. megaterium* ATCC 14581 was successfully engineered to overproduce 2’-FL, a valuable human milk oligosaccharide, through a series of genetic modifications and metabolic pathway optimizations. This work underscores the feasibility of using GRAS strains for the production of oligosaccharides, paving the way for safer and more efficient methods in biotechnological applications. Future studies could explore additional genetic modifications and optimization of fermentation conditions of the strain to further enhance 2’-FL yield and scalability.

**Supplementary Information:**

The online version contains supplementary material available at 10.1186/s12934-024-02620-w.

## Introduction

Human Milk Oligosaccharides (HMOs) are a fascinating group of sugars, predominant in human breast milk with over 200 unique structures. Their concentration ranges from 5 to 15 g/L, significantly surpassing the levels found in the milk of other mammals [1–5]. Among the oligosaccharides, roughly 77% are fucosylated and 28% are sialylated, including key structures like 2’-fucosyllactose (2’-FL) and 3-fucosyllactose (3-FL) [6]. These oligosaccharides stand out for their prebiotic effects to hinder pathogen adhesion in the intestines, and their role in modulating the immune system [7, 8]. Some are even absorbed by infants, potentially influencing immune functions. Especially, 2’-FL, a major component representing about 30% of the amount of total HMOs in human breast milk, has been used in various products [9, 10].

Methods for synthesizing 2’-FL range from extraction directly from breast milk to more intricate chemical or enzymatic approaches [11]. The chemical synthesis process, although explored, is hindered by a convoluted series of reactions that result in suboptimal yields and productivity. Enzymatic synthesis, on the other hand, faces challenges due to the high costs of the required substrates and limited productivity stemming from a lack of substrate specificity [11, 12]. Consequently, there has been a significant shift towards employing genetically engineered microorganisms as a means to produce 2’-FL [13]. This bioengineering approach has provided more effective production of 2’-FL compared to the traditional chemical and enzymatic methods [14–16].

Among the microorganisms, *Escherichia coli* has been extensively engineered for 2’-FL production, focusing on improving GDP-L-fucose biosynthesis and lactose utilization. For increasing intracellular GDP-L-fucose avaiability, overexpression of key genes from the *de novo* pathway, including *manB* (phosphomannomutase), *manC* (mannose-1-phosphate guanylyltransferase), *gmd* (GDP-mannose 4,6-dehydratase), and *wcaG* (GDP-L-fucose synthase), have employed [17]. Also, the salvage pathway was optimized by *fucI* gene (L-fucose isomerase) and *fucK* gene (L-fuculokinase) deletions and *fkp* gene (L-fucokinase/GDP-L-fucose phosphorylase) overexpression [18]. To resolve the issue of lactose utilization, the promoter of the lac operon was switched from P_lac_ to P_tac_ [19], or *lacZ* gene (β-galactosidase) deletion and *lacY* gene (lactose permease) overexpression was attempted [20, 21]. In *Bacillus subtilis*, the deletion of *ganA* and *yesZ* genes (β-galactosidase) was employed to prevent lactose degradation, and similar engineering strategy as in *E. coli* has been applied to increase the GDP-L-fucose pool [22]. Yeast species such as *Saccharomyces cerevisiae* or *Yarrowia lipolytica* have also employed with similar strategies to enhance 2’-FL production [23, 24].

Previous studies have explored microbial production of 2’-FL using various host strains, including GRAS (Generally Recognized as Safe) strains. The use of GRAS strains is advantageous due to their compliance with safety standards, making them suitable for commercial applications. Also, microbial synthesis of 2’-FL has predominantly utilized α-1,2-fucosyltransferases (α1,2-fucTs) derived from pathogenic microorganisms, including *Helicobacter pylori* and *E. coli* strains O128, O86, and O126 [12, 19, 25–29]. When using enzymes derived from non-GRAS strains, additional safety evaluations and regulatory approval are required. Therefore, it is important to screen GRAS strains as a 2’-FL production host and to identify α1,2-fucTs from GRAS strains [8, 24, 30–32].

In the present study, we utilized the GRAS strain *Priestia megaterium* ATCC 14581 (NCBI Accession No. NZ_CP009920.1) as the host organism for the production of 2’-FL. *P. megaterium* has been successfully used in various industrial applications, demonstrating its versatility and efficiency. For example, *P. megaterium* was employed for the production of vitamin B12 [33] and for heparosan biosynthesis, resulting in significant production levels (250 mg/L) [34]. In our previous study, we introduced the α-1,2-fucosyltransferase (α1,2-fucT), originally derived from another GRAS strain, *P. megaterium* (NCBI Accession No. NZ_NUWU0100000) [35]. In current study, the α1,2-fucT was introduced into the genome of *P. megaterium* ATCC 14581. Employing this host strain, along with the application of metabolic engineering techniques aimed at improving lactose utilization and GDP-L-fucose biosynthesis pathways, we achieved a 2’-FL production level comparable to our previous *E. coli*-based process, demonstrating the potential of *P. megaterium* as an alternative GRAS host for 2’-FL production. The importance of this research lies in its contribution to the metabolic engineering of a newly introduced GRAS strain in 2’-FL production, potentially facilitating broader applications in healthcare and infant nutrition.

## Materials and methods

### Bacterial strains and plasmids

*P. megaterium* ATCC 14581 (NCBI Accession No. NZ_CP009920.1) was used as a host strain for 2’-FL production. To engineer the host, available vector information was sourced from https://www.mobitec.com/. The vectors p3Stop1623-2RBShp and pMGBm19 were selected for the study. Both vectors were designed as shuttle vectors to facilitate gene manipulation. The vector p3Stop1623-2RBShp has RebU origin, while pMGBm19 originates from pBM100, inferring that two vectors can be used simultaneously. *P. megaterium* ATCC 14581 harboring p3Stop1623-2RBShp or pMGBm19 could have resistance to tetracycline or chloramphenicol, respectively. The co-insertion of both vectors was confirmed by their antibiotic resistance, thus affirming their simultaneous integration.

For gene cloning, *E. coli* Trans10 Chemically Competent Cell (Transgen Biotech, Beijing, China) was used. All plasmids used in this study were constructed through Gibson assembly cloning method. Primers for plasmid construction was listed in Supplementary Table 1. For construction of p3Stop-manA, vector, a strong promoter P_*29010*_, which was screened from *P. megaterium* ATCC 14581 as described in the following section and *manA* were amplified using primer sets of V_p3Stop_for and V_p3Stop_rev, p29010_manA_for and p29010_manA_rev, and manA_for and manA_rev, respectively. Transcriptional terminator RS07870 was located at downstream of *manA*. Three DNA fragments were ligated using NEBuilder HiFi DNA Assembly (New England Biolab, MA, USA). The genes (*manB* and *manC*) and their promoters were amplified with the primers listed in Supplementary Table 1, respectively. The DNA fragments including vector were ligated to construct p3Stop-manCA or p3Stop-manCBA. Likewise, the genes encoding lactose permeases, *lac12* from *Kluyveromyces lactis*, *lacY* from *P. megaterium* and *lacY* from *E. coli* were cloned in pMGBm19 plasmid, respectively, under P_*29010*_ promoter. The genes *lac12* and *lacY* from *E. coli* were synthesized by IDT (Integrated DNA Technologies, Iowa, USA) with codons optimized for *P. megaterium*. For construction of pMGB-lacYEc-Bm2FT, pMGB-lacYEc was used as a vector template. Vector was amplified using a primer set of V_pMGB_lacY_for and V_pMGB_lacY_rev, and the insert *futC* gene from *P. megaterium* (NCBI Accession No. NZ_NUWU0100000) [35] was amplified using primers of Bm2FT_for and Bm2FT_rev with RBS sequence. Two DNA fragments were ligated using NEBuilder HiFi DNA Assembly. To construct the vectors pMGB-lacYEc-Bm2FT-ndk, pMGB-lacYEc-Bm2FT-gmk, and pMGB-lacYEc-Bm2FT-xpt, the base vector pMGB-lacYEc-Bm2FT was linearized using the restriction enzyme SmaI (New England Biolab, MA, USA). Then, P_*29010*_ promoter and each gene (*ndk*, *gmk*, and *xpt*) were amplified, respectively, using primers listed in Supplementary Table S1. Linearized vector, amplified promoter DNA fragments, and amplified gene fragments were ligated using NEBuilder HiFi DNA Assembly. All primers used in this study were synthesized from Macrogen (Seoul, South Korea), and all genes were amplified using Q5 DNA polymerase (New England Biolab, MA, USA) Table 1.

**Table 1:** Bacterial strains and plasmids used in this study

Strains and plasmids	Description	Reference
**Strains**		
*E. coli* Trans10	Host for gene cloning, F- mcrA Δ(mrr-hsdRMS-mcrBC) φ80 lacZΔM15Δ lacX74 recA1 araΔ139Δ(ara-leu)7697 galU galKrpsL (StrR) endA1 nupG	Transgen Biotech (Beijing, China)
*Priestia megaterium*		
ATCC14581	*P. megaterium* ATCC 14581 wild type	ATCC (Manassas, USA)
BMZ	*P. megaterium* ATCC 14581 Δ*lacZ*	This study
BMZF	*P. megaterium* BMZ, BG04_4979::P_29010__*futC*, BG04_5297::P_29010__*futC*	This study
BMZF1	*P. megaterium* BMZF, harboring p3Stop-manA	This study
BMZF2	*P. megaterium* BMZF, harboring p3Stop-manCA	This study
BMZF3	*P. megaterium* BMZF, harboring p3Stop-manCBA	This study
BMZF4	*P. megaterium* BMZF (UV and NTG treatment), harboring p3Stop-*manCA*	This study
BMZF5	*P. megaterium* BMZF4, harboring pMGB*-*lac12Kl	This study
BMZF6	*P. megaterium* BMZF4, harboring pMGB*-*lacYBm	This study
BMZF7	*P. megaterium* BMZF4, harboring pMGB*-*lacYEc	This study
BMZF8	*P. megaterium* BMZF4, harboring pMGB*-*lacYEc*-*Bm2FT	This study
BMZF9	*P. megaterium* BMZF4, harboring pMGB*-*lacYEc-Bm2FT-ndk	This study
BMZF10	*P. megaterium* BMZF4, harboring pMGB*-*lacYEc-Bm2FT-gmk	This study
BMZF11	*P. megaterium* BMZF4, harboring pMGB*-*lacYEc-Bm2FT-xpt	This study
**Plasmids**		
p3Stop1623-2RBShp	P_*29010*_ promoters, RebU ori, Tet^r^	Lab stock
pMGBm19	P_*29010*_ promoters, pBM100 ori, Cm^r^	Lab stock
p3Stop-manA	p3Stop1623-2RBShp– P_*29010*_-*manA*	This study
p3Stop-manCA	p3Stop1623-2RBShp– P_*29010*_-*manC* -P_*29010*_*-manA*	This study
p3Stop-manCAB	p3Stop1623-2RBShp– P_*29010*_-*manC*-P_*29010*_*-manB*-P_*29010*_*-manA*	This study
pMGB-lac12Kl	pMGBm19 – P_*29010*_-*lac12* derived from *Kluyveromyces lactis*	This study
pMGB-lacYBm	pMGBm19 – P_*29010*_-*lacY* derived from *P. megaterium*	This study
pMGB-lacYEc	pMGBm19 – P_*29010*_-*lacY* derived from *Escherichia coli*	This study
pMGB*-* lacYEc- Bm2FT	pMGBm19 – P_*29010*_-*lacY* derived from *E. coli* -*futC*	This study
pMGB*-* lacYEc- Bm2FT-ndk	pMGBm19 – P_*29010*_- *lacY* derived from *E. coli- futC-* P_*29010*_*-ndk*	This study
pMGB*-* lacYEc- Bm2FT-gmk	pMGBm19 – P_*29010*_- *lacY* derived from *E. coli- futC-* P_*29010*_*-gmk*	This study
pMGB*-* lacYEc- Bm2FT-xpt	pMGBm19 – P_*29010*_- *lacY* derived from *E. coli- futC-* P_*29010*_*-xpt*	This study
pMGBm19-Cas12a	pMGBm19 – P_*lac*_-Cas12 from *Francisella* – P_*J23119*_-gRNA scaffold	This study

For *futC* gene encoding α1,2-fucT integration into genome, CRISPR-Cas12a system was applied. For expression of Cas12a and gRNA scaffold, pMBGm19 was used. Cas12a and gRNA scaffold were expressed under lac promoter and J23119 promoter, respectively, that plasmid was named as pMGBm19-Cas12a. Two pseudogenes, BG04_4979 and BG04_5297, were chosen as Bm2FT integration locus. gRNA sequences for targeting BG04_4979 and BG04_5297 (Table S1) were chosen using RGEN Tools (http://www.rgenome.net/cas-designer/). For gRNA of BG04_4979 cloning, pMGBm19-Cas12a was cut using restriction enzyme AarI (Thermo Fisher Scientific, Massachusetts, USA). The set of oligomers which targeted the BG04_4979 was annealed by incubating 90 °C for 4 min and then 70 °C for 10 min and cooling slowly to room temperature (0.1 °C decrease per 10 s). Cut vector and annealed gRNA were ligated T4 ligase (Thermo Fisher Scientific, Massachusetts, USA). For donor DNA cloning, homology arms of BG04_4979 with length of 800 bp and *futC* gene with the P_*29010*_ promoter were amplified with length of 800 bp. pMGBm19-Cas12a harboring gRNA was cut using restriction enzymes SalI and KpnI (New England Biolab, MA, USA). Vector and three inserts were ligated using NEBuilder HiFi DNA Assembly. The pMGBm19-Cas12a harboring gRNA and donor DNA was transformed into *P. megaterium*. The integration of *futC* gene was confirmed by colony PCR using primer set of 4979_int_confirm_for and 4979_int_confirm_rev. In the same way, *futC* gene with the P_*29010*_ promoter was integrated into BG04_5297.

### Mining of native promoter and terminator candidates

Candidate promoters and terminators for gene expression regulation in *P. megaterium* were selected through analysis of available transcriptome data. Datasets SRR6664739 and SRR6664740, obtained from *P. megaterium* cultures, were downloaded from the NCBI SRA database and were mapped to the *P. megaterium* genome sequence. Mapping was performed using CLC Genomics Workbench (Version 6.5.1) with the following parameters: mismatch cost: 2, insertion cost: 3, deletion cost: 3, length fraction: 0.9, similarity fraction: 0.9, and non-specific match: map randomly.

Readcounts were normalized to RPKM (Reads Per Kilobase Million), and genes in the top 5th percentile were identified for each condition: SRR6664739 (pH 7) and SRR6664740 (pH 4.5), respectively. A total of 193 genes were found in the top 5th percentile across both conditions. To ensure the inclusion of ribosome binding sequences, non-coding genes, genes within operons, and genes with less than 200 bp distance from their upstream genes were excluded. Following manual curation, 10 promoter sequences were selected for experimental validation.

For terminator sequence selection, the TransTermHP prediction tool was employed [36]. Predicted terminators were ranked by RPKM, and 10 palindromic sequences that exhibited sharp decreases in RNA expression profiles were chosen.

### Random mutagenesis and genome sequencing

To develop an improved host strain for increased 2’-FL production, we employed random mutagenesis using selectable markers. Initially, we utilized UV and NTG (N-methyl-N’-nitro-N-nitrosoguanidine) treatments to induce random mutations in a specially engineered strain with targeted gene deletions to boost 2’-FL production. The process began with UV irradiation followed by NTG treatment, resulting in a strain with enhanced 2’-FL output. The mutagenesis and selection procedure consisted of three stages. After each round of UV and NTG-induced mutagenesis, we picked all possible colonies and cultivated them to evaluate their 2’-FL production levels. Colonies with the highest productivity were selected for the next mutagenesis round. This iterative approach of mutagenesis, cultivation, and selection ultimately led to the development of a strain with significantly increased 2’-FL production. BMZF4 was selected as a production strain and its genome was sequenced using Illumina MiniSeq (Macrogen, Seoul, South Korea) and deposited in NCBI SRA (PRJNA1165678).

### Culture conditions and fermentation

During shake flask fermentation, Seed cultures were performed in a 15 mL test tube containing 3 mL of Luria-Bertani (LB) medium (1% tryptone, 0.5% yeast extract, 1% sodium chloride) with appropriate antibiotics (chloramphenicol 5 µg/mL) at 30 °C. The agitation speed was maintained at 250 rpm. The fermentation medium with the following composition was used: Yeast extract 3 g/L, (NH_4_)SO_4_ 1 g/L, KH_2_PO_4_ 3 g/L, K_2_HPO_4_ 12 g/L, NaCl 0.1 g/L, CaCl_2_-2H_2_O 0.015 g/L, FeSO_4_-7H_2_O 0.015 g/L, Thiamine 10 mg/L, 10 mL/L Trace Element Solution (Sodium citrate 20 g/L, MnCl_2_-4H_2_O 3.2 g/L, Zn(CH_3_COO)_2_-2H_2_O 1.68 g/L, CuCl_2_-2H_2_O 0.302 g/L, CoCl_2_-6H_2_O 0.534 g/L, H_3_BO_3_ 0.666 g/L, Na_2_MoO_4_-2H_2_O 0.534 g/L, 100 mL of 1 N HCl). Glucose, lactose and MgSO_4_-7H2O were sterilized separately and added to the sterilized shake flask to a final concentration 20, 15, 0.5 g/L, respectively. All engineered strains were cultivated on LB solid culture medium for overnight at 37 °C, and a single colony was inoculated into 3 mL liquid LB medium in 15 mL test tube at 37 °C and 220 rpm for 10–12 h.

The main cultures were further inoculated into 50 mL of the fermentation medium at the rate of 10% in a 250 mL baffled flask at 30 °C and 220 rpm for 72 h, and three replicates were set for each strain. Sampling was performed every 24 h for OD_600_, 2′-FL, carbon source, and by-products measurements. During fed-batch fermentation in 5 L jar fermentor, the fermentation medium used for the fed-batch culture consists of Yeast extract 8 g/L, (NH_4_)SO_4_ 2 g/L, KH_2_PO_4_ 10 g/L, K_2_HPO_4_ 2.5 g/L, NaCl 0.1 g/L, Threonine 0.5 g/L, CaCl_2_-2H_2_O 0.015 g/L, FeSO_4_-7H_2_O 0.015 g/L, Thiamine 10 mg/L, 10 mL/L Trace Element Solution (Sodium citrate 20 g/L, MnCl_2_-4H_2_O 3.2 g/L, Zn(CH_3_COO)_2_-2H_2_O 1.68 g/L, CuCl_2_-2H_2_O 0.302 g/L, CoCl_2_-6H_2_O 0.534 g/L, H_3_BO_3_ 0.666 g/L, Na_2_MoO_4_-2H_2_O 0.534 g/L, 100 mL 1 N HCl). Glucose (40 g/L), lactose (40 g/L), and MgSO₄-7 H₂O (1.2 g/L) were added to the medium after sterilized separately. Seed culture was carried out in a 250 mL flask containing 50 mL of LB medium with appropriate antibiotics (chloramphenicol 5 µg/mL, tetracycline 5 µg/mL) at 30 °C. The agitation speed was maintained at 250 rpm for 16 h. The seed culture (50mL) was inoculated to a 5 L Jar fermentor (CNS Bio Control & System, Daejeon, South Korea) with an initial 2.0 L fermentation medium. The pH was kept at 7.0–7.2 by adding 29% ammonia, and the temperature was maintained at 30 °C. The aeration rate and the agitation speed were 1.0 vvm and 900 rpm, respectively. Glucose was maintained at 5 ~ 10 g/L by feeding concentrated glucose (750 g/L), MgSO_4_-7H_2_O (15 g/L), and TMS (10mL/L). Lactose intermittently supplied around 20 to 30 g/L when it reached concentration between 10 and 20 g/L compared to the initial concentration. During the fermentation, cultivation was conducted using the pH-stat fed-batch mode, where carbon source feeding was controlled by monitoring pH fluctuations; when the pH increased by 0.05, a small amount of sugar was added to maintain optimal microbial activity without excessive sugar accumulation. All chemicals used for cultivation and fed-batch fermentation were purchased from Sigma-Aldrich (St. Louis, USA).

### Metabolite assays

Optical density measurements at 600 nm (OD_600_) were conducted using a DU730 spectrometer (Beckman Coulter, Brea, CA, USA). To halt enzymatic activities, samples from culture broths were subjected to a temperature of 98°C for a duration of 3 minutes. Subsequently, the supernatant was separated for analysis of extracellular metabolites following centrifugation at 27,670 rcf for a period of 10 minutes. The quantification of lactose, glucose and 2’-FL was performed through high-performance liquid chromatography (HPLC) (Waters 2695, Milford, MA, USA). The analysis employed an Rezex ROA Organic Acid H+ (8%) column (Phenomenx, USA), maintaining the column oven temperature at 75 °C. A mobile phase consisting of a 10 mM sulfuric acid solution was applied at a flow rate of 0.6 mL/min for the HPLC analysis. Standards for the calibration curve were sourced from Sigma-Aldrich (St. Louis, USA), ensuring the accuracy of metabolite quantification. The biosynthesis level of GDP-L-fucose was also measured using HPLC (Waters 2695, Milford, MA, USA) with a C18 column (Phenomenx, USA). The column oven temperature was maintained at 40 °C, and detection was performed at a wavelength of 254 nm. Two mobile phases were used: mobile phase A consisted of 20 mM triethylammonium acetate buffer adjusted to pH 6.0, and mobile phase B was acetonitrile.

## Results

### Advances in 2’-FL production via genetic engineering and precursor optimization

**Fig. 1:**
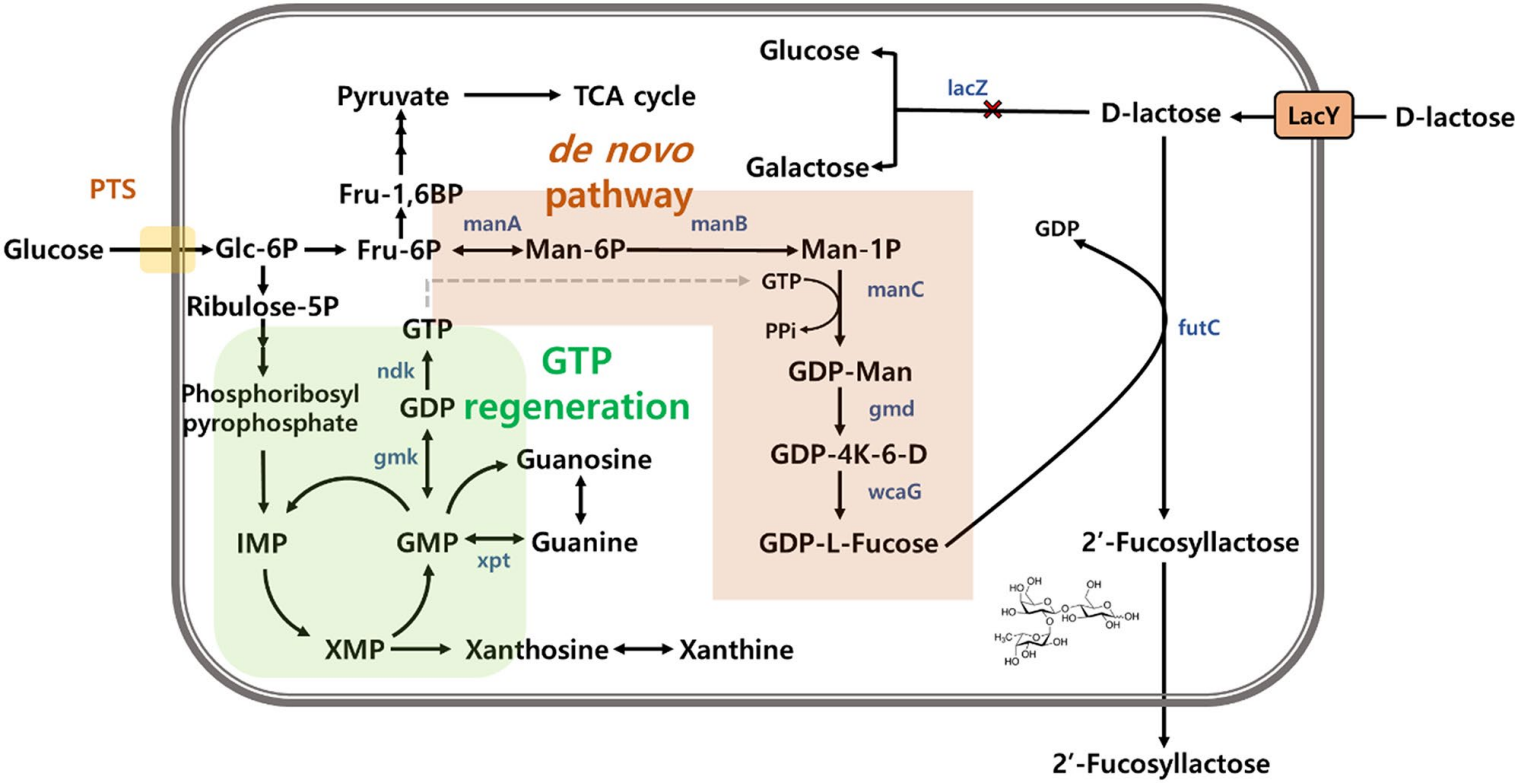
Biosynthesis pathway of 2’-Fucosyllactose in *P. megaterium* ATCC 14581. 2 genes (*manA*, *manC*) were overexpressed for the synthesis of GDP-L-fucose in this study. The *futC* gene from *P. megaterium*, encoding ⍺1,2-fucT, was inserted and overexpressed for the synthesis of 2’-FL. Glc-6P, Glucose-6-phosphate; Fru-6P, Fructose-6-phosphate; Fru-1,6BP, Fructose-1,6-bisphosphate; Man-6P, Mannose-6-phosphate; Man-1P, Mannose-1-phosphate; GDP-Man, GDP-Mannose; GDP-4 K-6-D, GDP-4-keto-6-deoxy-mannose; Fuc-1P, Fucose-1-phosphate; Fuculose-1P, Fuculose-1-phosphate; Ribulose-5P, Ribulose-5-phosphate; IMP, Inosine monophosphate; XMP, Xanthosine monophosphate; GMP, Guanosine monophosphate; GDP, Guanosine diphosphate; GTP, Guanosine triphosphate; *lacZ* encoding β-galactosidase; *lacY*,lactose permease; *manA*, mannose-6-phosphate isomerase; *manB*, phosphomannomutase); *manC*, mannose-1-phosphate guanylyltransferase; *gmd*, GDP-mannose 4,6-dehydrogenase; *wcaG*, GDP-L-fucose synthase; *futC*, α-1,2-fucosyltransferase; *ndk*, nucleoside diphosphate kinase; *gmk*, guanylate kinase; *xpt*, xanthine phosphoribosyltransferase

In this study, we performed various engineering strategies on *P. megaterium* ATCC 14581, a GRAS microorganism, to enhance 2’-FL production. Because lactose is a component of 2’-FL, lactose degradation, primarily mediated by the *lacZ* gene product, should be prevented. Therefore, deletion of the *lacZ* via homologous recombination was used to enhance lactose availability (Fig. 1). As depicted in Fig. S1, the wild-type strain displayed a blue color due to the native LacZ activity on X-gal, whereas the engineered *P. megaterium* strain with the *lacZ* deletion, BMZ, lacked this blue coloration, indicating the successful deletion of *lacZ* gene and retaining its original white phenotype.

To facilitate the production of 2’-FL in BMZ, *futC* gene encoding α1,2-fucT, from a non-pathogenic *P. megaterium* was introduced into BMZ strain. Because fucosyltransferase is generally known to be rate-limiting step in 2’-FL production [37, 38], we attempted to express this gene with a strong promoter. For this purpose, a screening of strong promoters was conducted within BMZ strain. As shown in Fig. S2, by fusing several candidate promoters with *sfGFP* encoding Superfolder Green Fluorescent Protein and selecting those that resulted in high fluorescence levels, we identified a native promoter, P_*29010*_ (Fig S1).

**Fig. 2:**
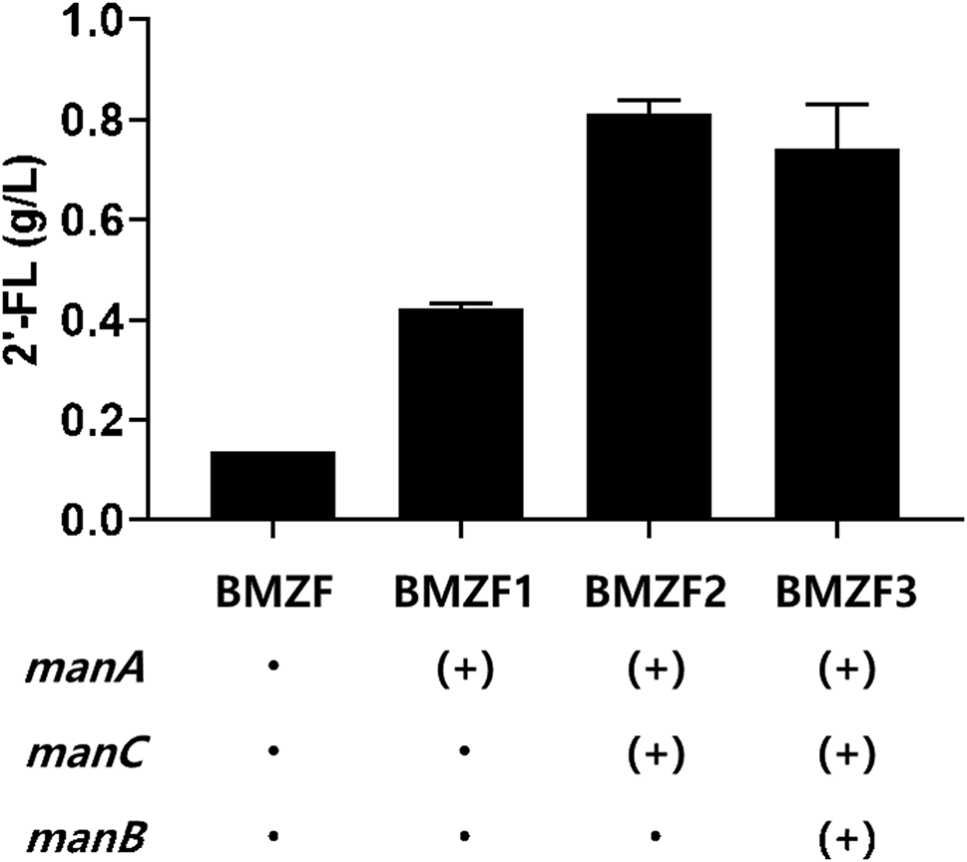
The production titer of 2’-FL in engineered *P. megaterium* ATCC 14581 strains. In all strains shown in this figure, *futC* gene was inserted and *lacZ* gene was deleted in their genomes. Additionally, *manA*, *manC*, and *manB*, were overexpressed to enhance the intracellular level of the precursor GDP-L-fucose. ‘(+)’ indicates the overexpressed genes in the strain

Additionally, we tested two different terminator sequences to determine which would result in higher 2’-FL production. Based on our findings, we selected the terminator from *BG04_RS07870* gene encoding GatB/YqeY domain-containing protein that significantly enhanced 2’-FL yield and used it for all subsequent constructions (Fig S1). The *futC* gene with the strong promoter P_*29010*_ and the BG04_RS07870 terminator was integrated into two pseudogene locations, *BG04_4979* and *BG04_5297*, using the CRISPR-Cas12 system. The resulting strain with a double-copy of the *futC* gene in its genome was named BMZF. The BMZF strain exhibited a 2’-FL production titer of 0.14 g/L (Fig. 2).

Next, to increase the intracellular concentration of GDP-L-fucose, a main precursor for 2’-FL synthesis, we decided to overexpress the *manA* gene, which is the first step from fructose-6-phosphate. The *manA* gene from *P. megaterium* was expressed under the control of a strong promoter, P_*29010*_, with a terminator RS07870. As a result, we observed a higher production of 2’-FL (0.42 g/L) in the strain with *manA* overexpression. Furthermore, we explored various combinations of genes involved in the *de novo* pathway (*manB*, *manC*, *gmd*, *wcaG*) to determine the most effective configuration for 2’-FL production. We initially overexpressed *manB* and *manC* genes sequentially after *manA*, each under the control of the P_*29010*_ promoter and followed by a terminator RS07870. As shown in Fig. 2, the highest 2’-FL production (0.81 g/L) was achieved when *manC* was overexpressed together with *manA*. Subsequently, we also overexpressed *gmd* and *wcaG* following *manA* and *manC*, but this did not result in increased 2’-FL production (data not shown). Through this process, we confirmed that the co-overexpression of *manA* and *manC* yields the highest 2’-FL production in our current strain, which was named BMZF2.

### Random mutagenesis for improvement of 2’-FL production

**Fig. 3:**
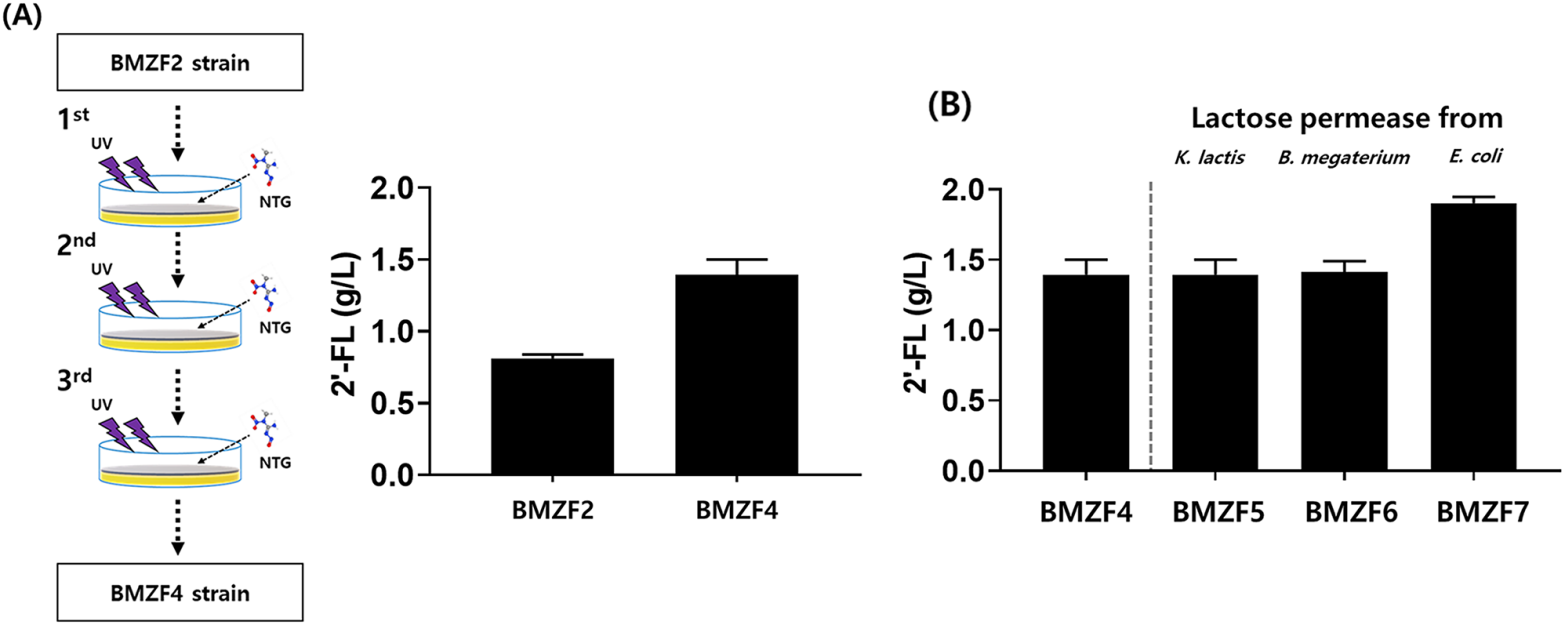
(**A**) Using UV and NTG treatments to induce random mutations, followed by selection with antimycin and 6’-azauracil, we identified the BMZF4 strain, which exhibited significantly increased 2’-FL production. This approach effectively enhanced the genetic diversity, allowing us to isolate a highly productive mutant strain. (**B**) Genes encoding lactose permeases derived from *K. lactis*, *P. megaterium*, and *E. coli* were overexpressed in vector form, leading to the creation of strains BMZF5 through BMZF7. The 2’-FL production levels of each of these strains were subsequently measured to evaluate the effectiveness of lactose permease overexpression in improving 2’-FL synthesis

To develop a host strain for increased 2’-FL production, random mutagenesis was carried out. In the initial phase of our study, we employed UV and NTG (N-methyl-N’-nitro-N-nitrosoguanidine) treatments to induce random mutagenesis in the engineered strain, BMZF2, aiming to improve 2’-FL production [39]. This approach involved exposure to UV irradiation, followed by NTG treatment, to enhance mutagenesis rate and, consequently, the strain’s 2’-FL productivity. Typically, 6-azauracil is used to inhibit GTP biosynthesis in *S. cerevisiae*. Since GTP plays a crucial role in RNA synthesis, reducing its levels can significantly impact cell viability [40, 41]. By adding 6’-azauracil to the growth medium and subsequently selecting the surviving colonies, we aimed to identify strains more adept at GTP synthesis. Additionally, antimycin was employed during random mutagenesis to obtain strains with a high NADPH pool for enhanced GDP-L-fucose biosynthesis, while 6’-azauracil was used as a selective marker, resulting in further enhancements. Random mutations were introduced through three successive stages using UV and NTG treatments. In each round, all possible colonies (about 300 colonies) were picked and their 2’-FL production levels were individually measured. The colonies that exhibited the highest 2’-FL productivity were selected for the next round of mutagenesis. Notably, our third-stage mutant strains showed substantial improvements in 2’-FL productivity. One of the strains, named as BMZF4, presented a promising advancement in the 2’-FL productivity achieving levels up to 1.3 g/L (Fig. 3A). Notably, BMZF4 demonstrated approximately a twofold increase (17.1 mg/L) in GDP-L-fucose levels compared to the BMZF2 strain, highlighting the enhanced metabolic capacity of BMZF4 for 2’-FL production (Fig S3). The genome of the BMZF4 strain was sequenced, and some of the mutations in the genome were listed in Supplementary Table 2.

### Optimization of lactose uptake through introduction of lactose permeases

To further increase 2’-FL production, we investigated the impact of different lactose permeases on lactose uptake. This involved the integration of lactose permease genes—*lac12* from *K. lactis*, and *lacY* from both *P. megaterium* and *E. coli*—into the multi-copy number plasmid pMGBm19. The implementation of this strategy yielded significant improvements in 2’-FL production, particularly when utilizing the *lacY* gene from *E. coli*, showing the highest production titer as depicted in Fig. 3B. This enhancement in lactose uptake, facilitated by the *E. coli*-derived *lacY* gene, the resulting strain BMZF7, significantly increased lactose availability within the cell, leading to a notable rise in 2’-FL synthesis. These results highlight the role of transporter in addressing substrate availability challenges.

**Fig. 4:**
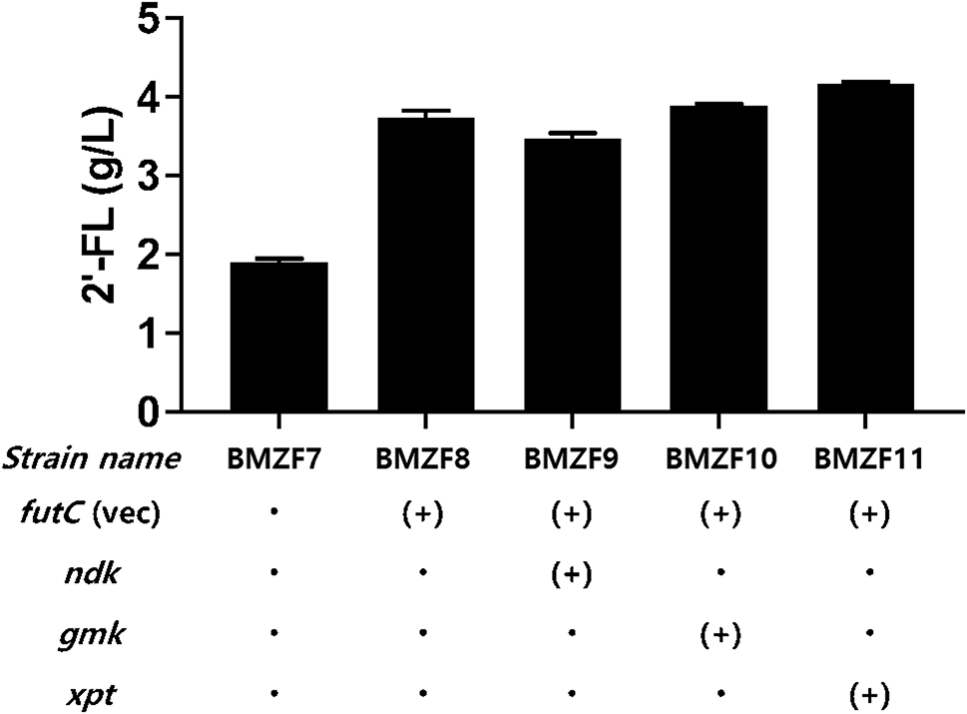
Effects of GTP regeneration pathway genes on 2’-FL production. In strains with enhanced lactose availability, further augmentation of 2’-FL production was pursued by co-expressing the *futC* gene in a vector alongside the *lacY* gene derived from *E. coli* (BMZF8). Additionally, the genes *ndk*, *gmk*, and *xpt* were individually overexpressed using vectors to enhance GTP regeneration. ‘(+)’ indicates the overexpression of genes by vectors

Following the ample supply of lactose, dramatic increase in 2’-FL production was attempted by overexpressing the key enzyme, α1,2-fucT, within the cell. The resulting strain, BMZF8, was meticulously designed for overexpression of the *futC* gene with alongside the *lacY* gene from *E. coli*. Remarkably, this approach resulted in 2-folds increase in 2’-FL production, culminating in an impressive titer of 3.7 g/L (Fig. 4). This substantial increase underscored the important role of optimizing both substrate availability and the concentration of key biosynthetic enzymes in enhancing 2’-FL yields.

Furthermore, additional engineering efforts were directed towards maintaining a high intracellular concentration of GDP-L-fucose. A crucial aspect of this strategy involved facilitating GTP regeneration, as GTP serves as a foundational substrate for GDP-L-fucose production. We overexpressed three genes from *E. coli*—*ndk*, *gmk*, and *xpt*—each playing a role in the GTP regeneration pathway. Among these, the overexpression of *xpt* gene was found to be the most effective, resulting in a 10% increase in 2’-FL production, reaching a new peak of 4.1 g/L (Fig. 4). The resulting strain was named BMZF11 and was used in subsequent fermentation experiments.

### Fed-batch fermentation for 2’-FL production

**Fig. 5:**
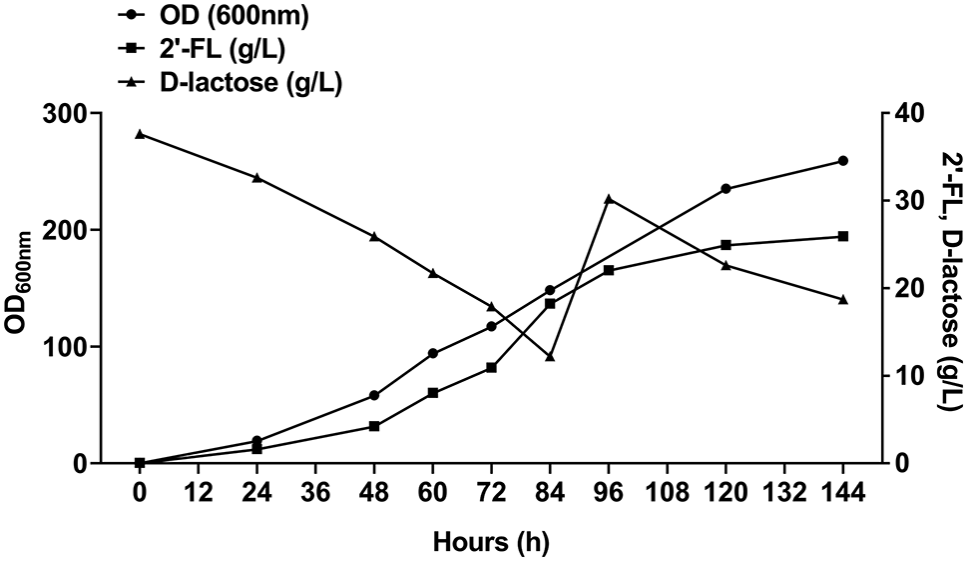
Results of 144 h fed-batch fermentation with 2 L working volume. Lactose (40 g/L) and glucose (20 g/L) were used for initial media. Glucose was maintained at 20 g/L, and a pH-stat mode was employed for its continuous supplementation. The 2’-FL overproducing strain, BMZF11, was used for 2’-FL synthesis. Black circle, cell growth; Black square, 2’-FL (g/L); Black triangle, D-lactose (g/L)

The production of 2’-FL was carried out in a 5 L fermenter with a working volume of 2 L. The pH was maintained between 7.0 and 7.2 through the addition of ammonium hydroxide (NH_4_OH). Glucose served as the primary carbon source for cellular growth, while lactose did not because of the *lacZ* deletion in the strain. Because lactose was used as a fucose receptor during fermentation, its concentration was monitored hourly to ensure it did not drop significantly, with an initial concentration set at 40 g/L. Additionally, glucose was maintained at 20 g/L, and a pH-stat mode was employed for its continuous supplementation. The engineered strain BMZF11, optimized for enhanced lactose utilization and increased intracellular GDP-L-fucose levels, was utilized for fermentation. This approach resulted in a biomass of 92.8 g dry cell weight (DCW)/L and a final 2’-FL concentration of 28.6 g/L, achieving a yield of 0.69 g/g lactose and 0.22 g/g glucose, which is significantly higher than the 0.26 g/g lactose yield observed in the previous flask cultivation. (Fig. 5). These findings illustrate that the strategic supplementation of approximately 130 g/L of total glucose, intermediate lactose feeding, effective regeneration of GTP, and directing metabolic flux towards the target biosynthetic pathway are crucial for the efficient production of 2’-FL.

## Discussion

The use of GRAS strains, such as *P. megaterium*, presents a promising biotechnological platform for the production of 2’-FL, a critical human milk oligosaccharide vital for infant gut microbiota development and immunity. *P. megaterium* offers several advantages, including its robust growth, high-cell density cultivation, and well-established safety profile, which make it particularly suitable for industrial-scale production [42, 43].

In this study, we strategically engineered *P. megaterium* ATCC 14581 by deleting the *lacZ* gene to prevent lactose degradation, thus enhancing lactose availability for 2’-FL production. We further introduced and overexpressed the *futC* gene encoding α1,2-fucT from *P. megaterium*. Additionally, we optimized the GDP-L-fucose biosynthesis pathway by overexpressing a combination of *manA* and *manC* genes. During the process, the identification and application of strong promoters (P_*29010*_) and terminators, which were previously not well characterized in *P. megaterium*, played a crucial role in enhancing gene expression and 2’-FL production. The success of using these genetic elements represents a significant advancement and is essential for *P. megaterium* to become a more efficient production host, similar to well-established strains like *E. coli* and *B. subtilis*.

We employed iterative rounds of random mutagenesis, facilitated by UV and NTG treatments, followed by rigorous selection processes, leading to the development of strains with significantly elevated 2’-FL productivity. The use of selectable markers like antimycin and 6’-azauracil further enhanced these efforts. Moreover, optimizing GTP regeneration by overexpressing *xpt* gene further boosted 2’-FL yields.

Despite these advancements, the 2’-FL production levels in our engineered *P. megaterium* strains (28.6 g/L) have not yet reached the levels reported in recent studies, such as the approximately 40 g/L production achieved using the GRAS strain *Y. lipolytica* [24]. This underscores the necessity for further modifications, which may include engineering against carbon catabolite repression (CCR) and enhancing GDP-L-fucose availability through targeted deletion of competing pathways [19, 44]. Additionally, similar to studies introducing *E. coli*’s SetA as a 2’-FL exporter, searching for the exporter in this strain should be considered for future work to enhance 2’-FL production [45]. Prior studies in other systems have demonstrated the importance of such modifications. By addressing these areas, we anticipate overcoming current limitations, thereby reinforcing the industrial viability of utilizing *P. megaterium* for the safe production of functionally important oligosaccharides.

## Conclusion

In conclusion, this study successfully demonstrates the metabolic engineering of the GRAS strain *P. megaterium* ATCC 14581 for enhanced production of 2’-FL, a key human milk oligosaccharide with significant applications in infant nutrition and gut microbiota development. By deleting the *lacZ* gene to prevent lactose degradation and introducing and overexpressing the *futC* gene encoding α1,2-fucT from *P. megaterium*, along with strategic enhancements in lactose utilization and GDP-L-fucose biosynthesis, we significantly improved 2’-FL yields. Further yield improvements were achieved through random mutagenesis and the optimization of metabolic pathways for lactose availability and GTP regeneration. These modifications resulted in a significant 2’-FL yield of 28.6 g/L during fed-batch fermentation. This demonstrates the effectiveness and potential of our metabolic engineering strategies applied to *P. megaterium*.

## Electronic Supplementary Material

Below is the link to the electronic supplementary material.

Supplementary Material 1

## Data Availability

No datasets were generated or analysed during the current study.

## Abbreviations

2’-FL: 2’-Fucosyllactose
HMO: Human Milk Oligosaccharide
GRAS: Generally Recognized As Safe
CRISPR: Clustered regularly interspaced short palindromic repeats
