# Supplementary material for: Metabolic engineering of Priestia megaterium for 2’-fucosyllactose production
Source: Microb Cell Fact. 2025 Jan 4;24:2. doi: 10.1186/s12934-024-02620-w (PMC11699682; doi:10.1186/s12934-024-02620-w)
Supplement: Supplementary file 1 — Supplementary Material 1 [file 12934_2024_2620_MOESM1_ESM.docx]

Supporting Information

Metabolic engineering of *Priestia megaterium* for 2’-fucosyllactose production

Bu-Soo Park^1,2†^_,_ Jihee Yoon^2,†^, Jun-Min Lee^1,†^, Sang-Hyeok Cho^3,4^, Yoojeong Choi^3,4^, Byung-Kwan Cho^3,4,5,^*, Min-Kyu Oh^1,^*

^1^ Department of Chemical & Biological Engineering, Korea University, Seoul 136-763, Korea

^2^ Samyang Corp. 295 Pangyo-ro, Bundang-gu, Seongnam-si, Gyeonggi-do 13488, Republic of Korea

^3^ Department of Biological Sciences, Korea Advanced Institute of Science and Technology, Daejeon, 34141, Republic of Korea

^4^ KAIST Institute for the BioCentury, Korea Advanced Institute of Science and Technology, Daejeon, 34141, Republic of Korea

^5^ Graduate School of Engineering Biology, Korea Advanced Institute of Science and Technology, Daejeon, 34141, Republic of Korea

† These authors contributed equally to this work.

^*^ Corresponding author. **Min-Kyu Oh** ([mkoh@korea.ac.kr](mailto:mkoh@korea.ac.kr)), **Byung-Kwan Cho** (bcho@kaist.ac.kr)

**Table S1.** Oligomers used in this study

| **Vector** | **Primer** | **Sequence (5’ → 3’)** |
| --- | --- | --- |
| p3Stop-manA | V_p3Stop_for | ACCTCGCGAACGGATTCAC |
|  | V_p3Stop_rev | TCTTAAGGAACGTACAGACGG |
|  | P29010_manA_for | CGTCTGTACGTTCCTTAAGATCCAACTGCTTTCATAATAAG |
|  | P29010_manA_rev | CTTTTTTCATAACTTTCACTCTCCCTATTAATC |
|  | manA_for | AGTGAAAGTTATGAAAAAAGCACCTATTTTTTTAG |
|  | manA_rev | GGTGAATCCGTTCGCGAGGTCACCTTACACAAGGTAAGGTGTTAGATTTTTGCCATAGCTC |
| p3Stop-manCA | V_p3Stop-manA_for | TCCAACTGCTTTCATAATAAG |
|  | V_p3Stop-manA_rev | TCTTAAGGAACGTACAGAC |
|  | P29010_manC_for | TAAGCCGTCTGTACGTTCCTTAAGATCCAACTGCTTTCATAATAAG |
|  | P29010_manC_rev | TACAAGTTTCATAACTTTCACTCTCCCTATTAATC |
|  | manC_for | GAGAGTGAAAGTTATGAAACTTGTATTGTTATCGG |
|  | manC_rev | TTATTATGAAAGCAGTTGGACACCTTACACAAGGTAAGGTGTTATAAAATCACCTGCGG |
| p3Stop-manCAB | V_p3Stop-manA_for | TCCAACTGCTTTCATAATAAG |
|  | V_p3Stop-manA_rev | TCTTAAGGAACGTACAGAC |
|  | P29010_manC_for | TAAGCCGTCTGTACGTTCCTTAAGATCCAACTGCTTTCATAATAAG |
|  | P29010_manC_rev | TACAAGTTTCATAACTTTCACTCTCCCTATTAATC |
|  | manC_P29010_for | GAGAGTGAAAGTTATGAAACTTGTATTGTTATCGG |
|  | manC_P29010_rev | GAAAGCAGTTGGACACCTTACACAAGGTAAGGTGTTATAAAATCACCTGCGG |
|  | P29010_manB_for | GTGATTTTATAACACCTTACCTTGTGTAAGGTGTCCAACTGCTTTCATAATAAG |
|  | P29010_manB_rev | AATGGCTTTCAAAACTTTCACTCTCCCTATTAATC |
|  | manB_for | GAGAGTGAAAGTTTTGAAAGCCATTATACTTGC |
|  | manB_rev | AATTCTTATTATGAAAGCAGTTGGAAGGGTAACTATTGCCGGATCCACCTTACACAAGGTAAGGTGTTACACTCTTTGATACTCTTG |
| pMGB-lac12Kl | V_pMGB_for | GTCCAAACTAGTTCGAAGATCTC |
|  | V_pMGB_rev | TTACGCATCTGTGCGGTATTTC |
|  | P29010_lac12Kl_for | TGTGAAATACCGCACAGATGCGTAATCCAACTGCTTTCATAATAAG |
|  | P29010_lac12Kl_rev | GTGATCAGCCATAACTTTCACTCTCCCTATTAATC |
|  | Lac12_for | GAGAGTGAAAGTTATGGCTGATCACTCTTCTTC |
|  | Lac12_rev | CGGAGATCTTCGAACTAGTTTGGACCACCCTTACACAAGGTAAGG |
| pMGB-lacYBm | V_pMGB_for | GTCCAAACTAGTTCGAAGATCTC |
|  | V_pMGB_rev | TTACGCATCTGTGCGGTATTTC |
|  | P29010_lacYBm_for | TGTGAAATACCGCACAGATGCGTAATCCAACTGCTTTCATAATAAG |
|  | P29010_lacYBm_rev | TGAACTTTTCATAACTTTCACTCTCCCTATTAATC |
|  | LacYBm_for | GAGAGTGAAAGTTATGAAAAGTTCAAAAAGTTTATATTG |
|  | LacYBm_rev | CGGAGATCTTCGAACTAGTTTGGACCACCTTACACAAGGTAAGGTGCTATATAATCTGCGTGTTTTC |
| pMGB-lacYEc | V_pMGB_for | GTCCAAACTAGTTCGAAGATCTC |
|  | V_pMGB_rev | TTACGCATCTGTGCGGTATTTC |
|  | P29010_lacYEc_for | TGTGAAATACCGCACAGATGCGTAATCCAACTGCTTTCATAATAAG |
|  | P29010_lacYEc_rev | CAAGTAGTACATAACTTTCACTCTCCCTATTAATC |
|  | LacYEc_for | GAGAGTGAAAGTTATGTACTACTTGAAAAACACAAAC |
|  | LacYEc_rev | CGGAGATCTTCGAACTAGTTTGGACCACCTTACACAAGGTAAGGTGTTAAGCAACTTCATTCACTTG |
| pMGB-lacYEc-Bm2FT | V_pMGB_lacY_for | GTCCAAACTAGTTCGAAG |
|  | V_pMGB_lacY_rev | TTAAGCAACTTCATTCACTTG |
|  | Bm2FT_for | AAGTGAATGAAGTTGCTTAAAAAATTTAATAGGGGGTAACATGCAATGAAAATAGTACAAATAAGTAGTG |
|  | Bm2FT_rev | ATCTTCGAACTAGTTTGGACCATTAAAAAAGCATCCTAATAGATGGGATGCTTTTTTAATGTCAATAAACTATCCAGCC |
| pMGB- lacYEc-Bm2FT -ndk | P29010_ndk_for | TAGTTCGAAGATCTCCGGAGCTCCCTCCAACTGCTTTCATAATAAG |
|  | P29010_ndk_rev | TTTTTGTATCATAACTTTCACTCTCCCTATTAATC |
|  | ndk_for | GAGAGTGAAAGTTATGATACAAAAAACGTTTTTAATG |
|  | ndk_rev | AATATAAATGACTCTAGAGGATCCCCACCTTACACAAGGTAAGGTGGGTACCTTAGTAAATCCAAGCGTC |
| pMGB- lacYEc-Bm2FT -gmk | P29010_gmk_for | TAGTTCGAAGATCTCCGGAGCTCCCTCCAACTGCTTTCATAATAAG |
|  | P29010_gmk_rev | TCTTTCAATCATAACTTTCACTCTCCCTATTAATC |
|  | gmk_for | GAGAGTGAAAGTTATGATTGAAAGAGGTTTATTAATTG |
|  | gmk_rev | AATATAAATGACTCTAGAGGATCCCCACCTTACACAAGGTAAGGTGGGTACCTTATTCTACCTCCAGCATTC |
| pMGB- lacYEc-Bm2FT -xpt | P29010_xpt_for | TAGTTCGAAGATCTCCGGAGCTCCCTCCAACTGCTTTCATAATAAG |
|  | P29010_xpt_rev | CAAAGCTTTCATAACTTTCACTCTCCCTATTAATC |
|  | xpt_for | GAGAGTGAAAGTTATGAAAGCTTTGCAAAAC |
|  | xpt_rev | AATATAAATGACTCTAGAGGATCCCCACCTTACACAAGGTAAGGTGGGTACCTTAAACCTCCACTGTTGG |
| pMGBm19-Cas12a – 4979 – Bm2FT | gRNA_4979_sense | AGATTGGGCACAATACATCCAAGA |
|  | gRNA_4979_anti | AGACTCTTGGATGTATTGTGCCCA |
|  | LHR_4979_for | GTGGTGAATCCGTTAGCGAGGTGCGCTCGGTGCGATTTTGTTTTTC |
|  | LHR_4979_rev | GGTATTTCACACCAAATTCGGCACCTCCAATTATTAC |
|  | 4979_Bm2FT_for | GGTGCCGAATTTGGTGTGAAATACCGCACAG |
|  | 4979_Bm2FT_rev | AAATCTAATTTTGATCTTCGAATCAATAAACTATCCAG |
|  | RHR_4979_for | TGATTCGAAGATCAAAATTAGATTTACCGATTGGC |
|  | RHR_4979_rev | CTAGCTTTAATGCGGTAGTTGGTACAACGAACGGTCAAAGTCAG |
| Bm2FT_integration_confirm_4979 | 4979_int_confirm_for | ACTTGAACGCATTCCACTCA |
|  | 4979_int_confirm_rev | TGACCAAGAAACAGCGGATA |
| pMGBm19-Cas12a – 5297 – Bm2FT | gRNA_5297_sense | AGATAGGAACAAGGAGGGGGATAT |
|  | gRNA_5297_anti | AGACATATCCCCCTCCTTGTTCCT |
|  | LHR_5297_for | GTGGTGAATCCGTTAGCGAGGTGCGTAGTACTTGGTGGGTTTC |
|  | LHR_5297_rev | GGTATTTCACACCACTTTCTCCCCTTTACTTTTG |
|  | 5297_Bm2FT_for | AGGGGAGAAAGTGGTGTGAAATACCGCACAG |
|  | 5297_Bm2FT_rev | TGCTCTTCCTTTGATCTTCGAATCAATAAACTATCCAG |
|  | RHR_5297_for | TGATTCGAAGATCAAAGGAAGAGCAAGCGAC |
|  | RHR_5297_rev | CTAGCTTTAATGCGGTAGTTGGTACTCAAGGTTATTCCTTGGAG |
| Bm2FT_integration_confirm_5297 | 5297_int_confirm_for | AAGTCAACGCTTCCCTGAAA |
|  | 5297_int_confirm_rev | TTGAGCACAGCTCCCTTTTT |

**Table S2. Mutations in the BMZF4 strain compared to BMZF2**

| **Position** | **Reference** | **Alternate** | **Mutation type** | **Genes** | **Residue change** |
| --- | --- | --- | --- | --- | --- |
| **24310** | **C** | **T** | **Synonymous** | ***cysI*** | **D231D** |
| **24828** | **G** | **A** | **Non-synonymous** | ***cysI*** | **D59N** |
| **27305** | **G** | **A** | **Intergenic** |  |  |
| **33134** | **C** | **T** | **Non-synonymous** | ***BG04_42*** | **A34V** |
| **149043** | **G** | **A** | **Non-synonymous** | ***zwf*** | **G298E** |
| **232510** | **C** | **T** | **Synonymous** | ***BG04_282*** | **F159F** |
| **258965** | **C** | **T** | **Non-synonymous** | ***fbp*** | **L440F** |
| **394263** | **C** | **T** | **Non-synonymous** | ***BG04_475*** | **S242F** |
| **430820** | **G** | **A** | **Intergenic** |  |  |
| **432402** | **G** | **A** | **Non-synonymous** | ***ftsW*** | **G251R** |
| **455139** | **C** | **T** | **Intergenic** |  |  |
| **663237** | **C** | **T** | **Synonymous** | ***BG04_782*** | **G214G** |
| **1001320** | **C** | **T** | **Non-synonymous** | ***BG04_1096*** | **L61F** |
| **1008120** | **C** | **T** | **Non-synonymous** | ***BG04_1104*** | **A181V** |
| **1010390** | **C** | **T** | **Non-synonymous** | ***filL*** | **L226F** |
| **1609056** | **C** | **T** | **Non-synonymous** | ***ytaF*** | **A10V** |
| **1627513** | **C** | **T** | **Synonymous** | ***BG04_1769*** | **V318V** |
| **1681812** | **G** | **A** | **Intergenic** |  |  |
| **1695881** | **C** | **T** | **Non-synonymous** | ***BG04_1834*** | **L247F** |
| **1695991** | **C** | **T** | **Non-synonymous** | ***BG04_1834*** | **A210V** |
| **2007403** | **G** | **A** | **Synonymous** | ***rpoE*** | **G74G** |
| **2619849** | **C** | **T** | **Synonymous** | ***citN*** | **H327H** |
| **2647526** | **G** | **A** | **Synonymous** | ***BG04_2829*** | **Q97Q** |
| **2728093** | **C** | **T** | **Synonymous** | ***aceA*** | **Y61Y** |
| **2988917** | **G** | **A** | **Non-synonymous** | ***ssuD*** | **G50R** |
| **3144307** | **C** | **T** | **Non-synonymous** | ***BG04_3313*** | **P296S** |
| **3212591** | **C** | **T** | **Intergenic** |  |  |
| **3262444** | **G** | **A** | **Non-synonymous** | ***BG04_3414*** | **R47K** |
| **3401054** | **G** | **A** | **Synonymous** | ***mtnA*** | **E331E** |
| **3413776** | **G** | **A** | **Non-synonymous** | ***BG04_3556*** | **D284N** |
| **3457011** | **G** | **A** | **Non-synonymous** | ***ptsI*** | **G338D** |
| **3457289** | **G** | **A** | **Non-synonymous** | ***ptsI*** | **V431I** |
| **3629050** | **C** | **T** | **Non-synonymous** | ***BG04_3776*** | **L403F** |
| **3629508** | **G** | **A** | **Synonymous** | ***BG04_3776*** | **K555K** |
| **3670711** | **G** | **A** | **Non-synonymous** | ***BG04_3821*** | **G57D** |
| **4108941** | **C** | **T** | **Non-synonymous** | ***zwf*** | **S3F** |
| **4156328** | **C** | **T** | **Synonymous** | ***proC*** | **L250L** |
| **4595514** | **G** | **A** | **Non-synonymous** | ***BG04_4772*** | **G323S** |
| **4772363** | **C** | **T** | **Non-synonymous** | ***pbuX*** | **S12L** |
| **4772616** | **C** | **T** | **Synonymous** | ***pbuX*** | **S96S** |
| **4881019** | **G** | **A** | **Synonymous** | ***BG04_5089*** | **K383K** |
| **4881159** | **G** | **A** | **Non-synonymous** | ***BG04_5089*** | **A337T** |
| **5287592** | **C** | **T** | **Synonymous** | ***BG04_5526*** | **F306F** |


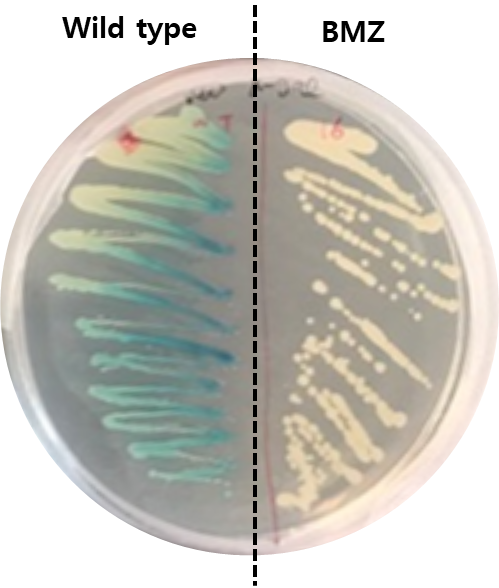


**Fig S1.** Identification and deletion of the *lacZ* gene in *P. megaterium* ATCC 14581. The genome sequence analysis pinpointed the *lacZ* gene involved in lactose degradation. Following homologous recombination, *lacZ* gene was successfully deleted, as evidenced by the color change observed on X-gal plates: the wild-type strain turned blue, indicating LacZ activity, while the engineered BMZ strain remained white, confirming the deletion of *lacZ* gene and preservation of its original phenotype.


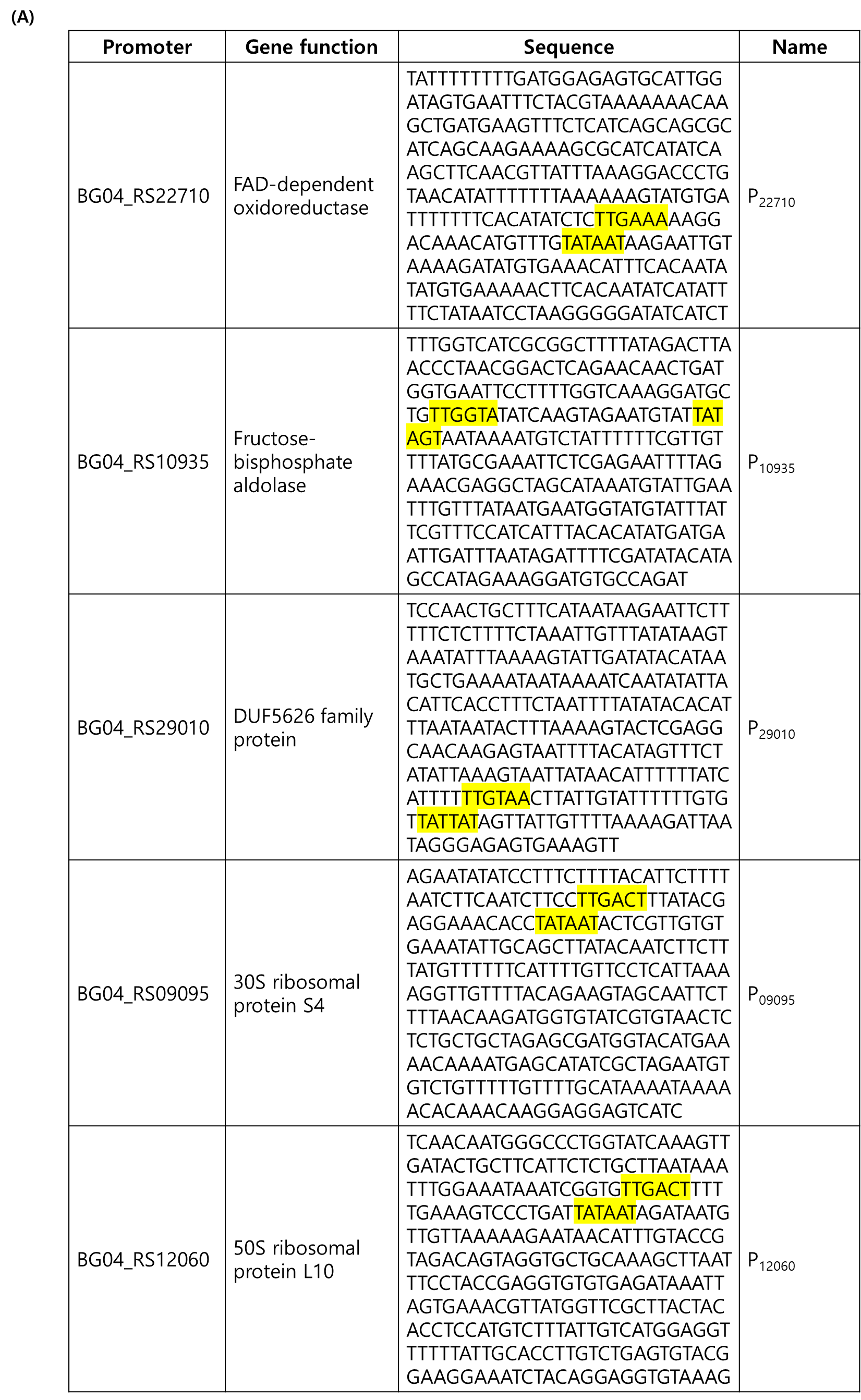


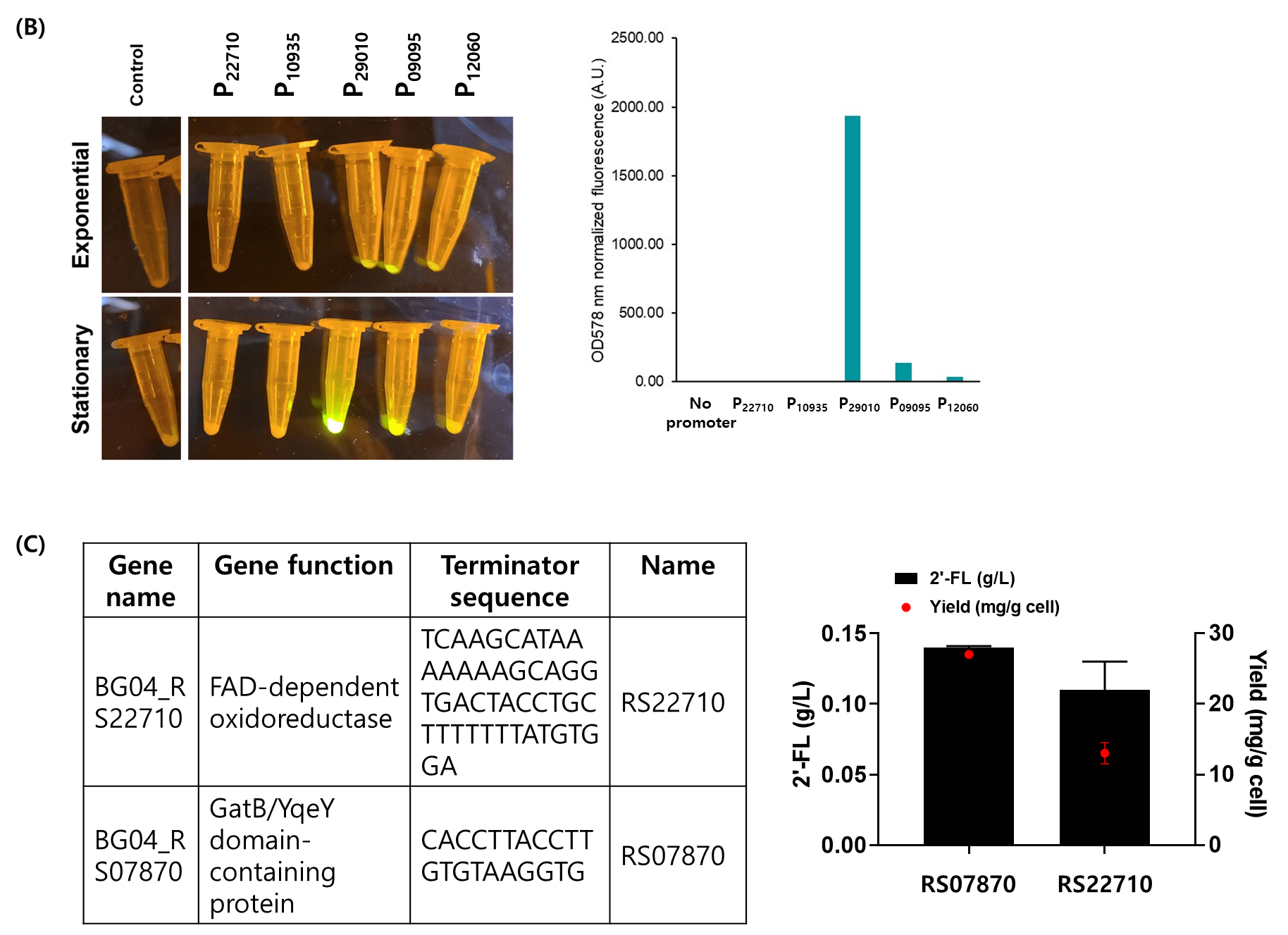


**Fig S2.** (A) List of five candidate promoters utilizable in *P. megaterium*, roles of the genes located downstream of each promoter, and the naming of these promoters. The 300 bp upstream region of the genes was utilized. The yellow highlighted regions were predicted to correspond to the -10 and -35 regions, respectively. (B) Identification of strong promoters for *futC* gene expression in *P. megaterium* ATCC 14581. Several candidate promoters were fused with the *sfGFP* gene encoding Superfolder Green Fluorescent Protein, and their activity was assessed based on fluorescence intensity. Among these, the native promoter P_29010_ from plasmid pBMV4, naturally occurring in *P. megaterium* ATCC 14581, demonstrated the highest fluorescence, indicating its strong expression capabilities. The figure displays the comparative fluorescence levels of five tested promoters, highlighting P_29010_ as the most effective. (C) To identify effective terminators within *P. megaterium*, we applied terminator prediction to various genes. The results indicated that the RS22710 and RS07870 terminators were the most effective for 2'-FL production. Consequently, for the construction of vectors involved in gRNA and 2'-FL production, we selected and used the RS07870 terminator sequence due to its superior performance.

**Fig S3.** Through UV and NTG treatments combined with antimycin, strains with enhanced GTP biosynthesis and elevated NADPH pools were selected. The BMZF4 strain demonstrated higher 2’-FL production compared to BMZF2, which is likely due to a twofold increase in GDP-L-fucose levels observed in BMZF4. This comparison between BMZF2 and BMZF4 highlights the impact of increased GDP-L-fucose availability on enhancing 2'-FL productivity.
